# Home Respiratory Polygraphy and Spirometry in Normal Weight and Children with Obesity Suspected for Obstructive Sleep Apnea Syndrome: Are There Any Associations?

**DOI:** 10.1155/2023/1532443

**Published:** 2023-01-31

**Authors:** Regula Corbelli, Constance Barazzone, Carole Grasset Salomon, Maurice Beghetti, Albane B. R. Maggio

**Affiliations:** ^1^Pediatric Pulmonary Unit, Division of Pediatric Specialties, Department of Pediatrics, Gynecology and Obstetrics, Geneva University Hospitals and University of Geneva, Switzerland; ^2^Pediatric Research Platform, Department of Pediatrics, Gynecology and Obstetrics, Geneva University Hospitals and University of Geneva, Switzerland; ^3^Pediatric Cardiology Unit, Division of Pediatric Specialties, Department of Pediatrics, Gynecology and Obstetrics, Geneva University Hospitals and University of Geneva, Switzerland; ^4^Pediatric Obesity Consultation, Division of Pediatric Specialties, Department of Pediatrics, Gynecology and Obstetrics, Geneva University Hospitals and University of Geneva, 1211 Geneva 14, Switzerland

## Abstract

**Aim:**

It is known that children and adolescents with obesity are more prone to obstructive sleep apnea syndrome (OSAS) and that their lung function may show some disturbance. Literature is scarce about potential associations; therefore, we aimed to study the relationship between OSAS, lung function, and adiposity in a population of children suspected of OSAS. *Material and Methods*. We performed home respiratory polygraphy and spirometry in all subjects. The relationships between body mass index *z*-score (zBMI), polygraphy, and spirometry data were analyzed.

**Results:**

We recruited 81 subjects aged between 5 and 16 years, 63% being obese. 43.2% of subjects were diagnosed with OSAS (32.1% mild, 4.9% moderate, and 6.2% severe). We found no correlation between respiratory polygraphy and the zBMI. The mean spirometric value FEV_1_, FVC, and FEV_1_/FVC ratio *z*'s were normal in all subjects, whereas FVC *z*'s and FEV_1_/FVC ratio *z*'s were significantly positively related for obesity and negatively for normal weight (*p* < 0.05). FEV_1_*z*'s was inversely correlated to the percentage of analyzed time passed below 90% of SpO_2_ (*r* = −0.224, *p* = 0.044). All subjects with FEV_1_ (*n* = 8) and/or FVC (*n* = 9) *z*'s below the lower limit for normal (LLN) had an AHI ≥ 1 (FEV_1_: *p* = 0.001; FVC: *p* < 0.001), especially subjects with normal weight (FEV_1_: *p* = 0.003; FVC: *p* = 0.010).

**Conclusion:**

When comparing normal-weight children and adolescents with obesity, the prevalence of OSAS but not spirometric values was strongly related to BMI *z*-score, probably because obesity engenders advanced puberty and an accelerated growth spurt. FEV_1_ was more frequently <LLN in normal-weight children, while obese subjects presented low FEV_1_/FVC ratio *z*'s and FEF_25-75%_*z*'s. Moreover, all subjects with abnormal spirometric values were suffering from at least mild OSAS, again more frequently in normal-weight subjects.

## 1. Introduction

Features of obstructive sleep-disordered breathing (SDB) observed in children and adolescents stand out from adults in terms of etiology, adenotonsillar hypertrophy being the most frequent, symptoms (nocturnal enuresis; behavioral, learning, and integrational problems; and hyperactivity), and treatment (medical or surgical ears, nose, and throat (ENT) interventions) [1]. Upper airway dysfunctions manifest with snoring and/or augmented respiratory effort, due to increased resistance in the upper airways and/or pharyngeal collapse during sleep. SDB is frequently divided into 4 clinical entities: primary snoring, upper airway resistance syndrome, obstructive hypoventilation, and obstructive sleep apnea-hypopnea syndrome (OSAS) [1]. OSAS is defined as obstructive sleep apnea associated with daytime symptoms. Therefore, OSAS is suspected in the presence of symptoms such as frequent snoring, witnessed respiratory pauses during sleep, fatigue during daytime, and, typically in children, hyperactivity. Severe OSAS might be suspected if a child sleeps in a seated position or with hyperextension of the neck or in the presence of enuresis. OSAS is frequent in overweight or underweight children, and typical clinical signs are adenotonsillar hypertrophy and facial abnormalities. Prevalence of habitual snoring in children is reported as 7.5% and OSAS between 1 and 4% [2], but this last can reach 13% in unselected obese children and as much as 59% in those with symptoms suggestive of sleep-disordered breathing, depending on different definitions and cut-offs used to define obesity and to diagnose OSAS [3].

Concerning lung function, some studies found that lung volumes might be lower in children with obesity due to functional restrictive syndrome [4], but other studies report opposite results [5, 6]. Values monitoring lower airway obstruction, such as FEV_1_/FVC ratio and FEF_25-75_, are not influenced by obesity [7–9]. However, whether there is a relationship between spirometric values and body mass index (BMI) is not properly established. Moreover, studies evaluating a relationship between pulmonary function tests and polysomnography or polygraphy in children with OSAS are scarce in the literature and show conflicting results [10–12]. Also, home respiratory polygraphy (HRP) is increasingly recognized as a reliable exam to detect OSAS in children [13, 14].

The first aim of this cross-sectional study was to compare home-based polygraphic and spirometric measurements between obese and normal-weight children and adolescents. The second aim was to evaluate the relationship between HRP and spirometric results and weight in this population of children and adolescents with signs and symptoms suspect for OSAS.

Indeed, if we could demonstrate a relationship between OSAS and abnormal lung function in children with obesity, we should consider investigating systematically this population with lung function testing.

## 2. Materials and Methods

### 2.1. Study Design and Subjects

This cross-sectional study was carried out at the University Children's Hospitals of Geneva, Switzerland. At the outpatient clinics of pulmonary, obesity and ENT patients aged 5 to 16 years with suspected obstructive sleep apnea-hypopnea syndrome (OSAS) were screened for inclusion. Exclusion criteria were (1) chronic pulmonary diseases (cystic fibrosis, interstitial lung disease, asthma, congenital pulmonary malformation, bronchiectasis, primary ciliary dysfunction, and bronchopulmonary dysplasia); (2) acute asthma exacerbation or uncontrolled asthma; (3) history of mechanical ventilation for lung diseases, tracheotomy, or pneumonia during the previous year; (4) neuromuscular diseases; (5) severe scoliosis; (6) syndromes with midface and ENT involvement; and (7) medications influencing sleep, blood pressure, or breathing patterns.

We categorized subjects according to their BMI *z*-score (zBMI): normal weight was defined as a zBMI < 1 SD, overweight as a zBMI between 1 and 2 SD, and obesity as a zBMI > 2 SD, based on the World Health Organization standards [15]. As there were no differences between overweight and obese subjects, we separated our subjects into 2 groups according to BMI: the “obese” group included those children who were overweight or obese; the “normal weight” group included the remaining subjects.

The study was approved by the Cantonal Ethics Committee (CER 12-221), and parental and child written informed consent was obtained.

### 2.2. Measurements

#### 2.2.1. Anthropometrics

We assessed body weight (kg) in light clothes (panties and tee-shirt) and height (cm) without shoes. Body mass index (BMI) was calculated as weight/height squared (kg·m^−2^), and *z*-scores (zBMI) were derived from the World Health Organization standards [15].

#### 2.2.2. Home Respiratory Polygraphy

Home respiratory polygraphy (HRP) was performed using the Embla® Embletta® GOLD portable sleep system. The detailed method description can be found in our previous publication [16].

Apnea was defined, according to pediatric scoring rules of the American Academy of Sleep Medicine 2012 [17], as a drop in the peak signal excursion of the nasal flow trace or belt sum trace (sum of thoracic and abdominal belts' traces) by ≥90% of the preevent baseline for at least the time equivalent to two respiratory cycles. When respiratory efforts were maintained, it was scored as obstructive apnea. When inspiratory efforts were absent and associated with ≥3% oxygen desaturation, it was scored as central apnea. Hypopnea was defined as a decrease of ≥30% in the amplitude of nasal flow trace or belt sum trace during the time equivalent to two respiratory cycles, associated with a drop in oxygen saturation of ≥3%.

The apnea-hypopnea index (AHI) and the obstructive apnea index (oAHI) were defined as the total number of respiratory events (apnea plus hypopnea) and the number of obstructive events, respectively, divided by the total analyzed study time in hours. The mean oxygen saturation was recorded, and the number of oxygen desaturations ≥ 3% divided by the total analyzed study time in hours was defined as the oxygen desaturation index (ODI). We also calculated the time passed with partial oxygen saturation below 90%, in the percentage of analyzed time (%time < 90%SpO_2_).

HRP tests were considered uninterpretable if, for technical reasons, the key signal (oxygen saturation) was absent or there were no reliable airflow and belt sum trace signals or if the analyzed time was less than 300 minutes.

OSAS was diagnosed if sleep-disordered breathing symptoms were present and the AHI (obstructive and central events) was ≥1/h [1]. We also used the following cut-offs to categorize OSAS severity: AHI < 1/h: no OSAS, 1 ≤ AHI < 5: mild OSAS, 5 ≤ AHI < 10: moderate OSAS, and AHI ≥ 10: severe OSAS [18].

#### 2.2.3. Spirometry

All subjects performed spirometry, without bronchodilator testing, using a handheld spirometer (MicroLoop from Micro Medical, Rochester, England, complying with 2005 American Thoracic Society/European Respiratory Society (ATS/ERS) standards). Spirometry was considered interpretable if fulfilling the ATS/ERS criteria [19]. We measured forced expiratory volume in 1 second (FEV_1_), forced vital capacity (FVC), and forced expiratory flow at 25-75% of forced vital capacity (FEF_25-75%_). We used prediction equations from the Global Lung Function Initiative (GLI 2012) [20] to calculate *z*-sores and percent predicted values for FEV_1_, FVC, FEV_1_/FVC ratio, and FEF_25-75%_. The GLI 2012 predicted values have been validated for multiethnic children [21] and Caucasian Australasians [22].

The ATS and ERS recommend an age-specific lower limit of normal (LLN) to be set at the 5^th^ percentile, corresponding to a *z*'s of -1.64 [23–25]. In contrast to % predicted, *z*'s are unbiased regarding age, height, sex, and ethnicity.

### 2.3. Statistical Analysis

Statistical analyses were performed using the SPSS software 25.0 (Chicago, IL). Descriptive analyses were performed using frequency distributions for the qualitative variables and mean and standard deviation (SD) for the quantitative variables. Statistical differences between normal weight and obese subjects were analyzed using independent Student's *t*-test and chi^2^ when appropriate. We evaluated the relationship between variables using Pearson coefficient correlation and regression analyses.

The sample size was calculated for the relationship between zBMI and FEV *z*'s, FEV/FVC *z*'s, and FVC *z*'s. Prior data indicate that the standard deviation of zBMI is 1, and the standard deviation of the regression errors will be 0.3. If the true slope of the line obtained by regressing the independent variable against zBMI is 0.1, we would need to study 73 subjects to be able to reject the null hypothesis that this slope equals zero with probability (power) 0.8. The Type I error probability associated with this test of this null hypothesis is 0.05.

Differences were considered significant if *p* < 0.05.

## 3. Results

### 3.1. Patient Characteristics

Out of the 91 subjects recruited, 81 had complete data for both home polygraphy and spirometry: 3 had to be excluded due to uninterpretable HRP and 7 due to uninterpretable pulmonary function tests. Therefore, we present the results of the 81 subjects. Our study population was only composed of Caucasians.

The obese group (*n* = 51) included 13 overweight and 38 obese subjects. There were 30 normal-weight children (zBMI < 1 SD). Characteristics of the subjects are presented according to their weight status in Table 1.

As expected, anthropometric characteristics between both groups differed significantly for weight and zBMI. Furthermore, in the obese group, children were older and taller (*p* < 0.001).

### 3.2. Home Respiratory Polygraphy

Based on the HRP recordings obtained, thirty-five (43.2%) out of the 81 subjects were diagnosed with at least mild OSAS (AHI ≥ 1), 32.1% (26/81) had mild OSAS, 4.9% (4/81) had moderate OSAS, and 6.2% (5/81) had severe OSAS.

Despite the fact that an AHI ≥ 1 seemed to be more frequent in children with obesity, the difference was not significant (obese: 47% vs. normal weight: 37%, *p* = 0.362), and there was no correlation between zBMI and AHI, oAHI, mean oxygen saturation, %time < 90%SpO_2_, or ODI (*p* > 0.05 for all).

### 3.3. Spirometry

Spirometry parameters for both groups are presented in Table 1. All mean values were within the normal range. However, some individuals presented spirometric values below the LLN (*z*′s < −1.64), mainly in normal-weight children (Table 1). Eight subjects had abnormal measures compatible with airway obstruction (FEV_1_/FVC ratio *z*′s < LLN and/or FEF_25-75_*z*′s < LLN), 7 of which were in the obese group. Only one young boy (8.3 years old) with a very high zBMI of 4.5 cumulated both abnormal measures.

When comparing the two groups for FEV_1_, FVC, and FEV_1_/FVC ratio *z*'s, we found higher FEV_1_ and FVC *z*'s and lower FEV_1_/FVC ratio *z*'s in the obese group. Indeed, several spirometric *z* data were correlated to zBMI (FEV_1_*z*'s: *r* = 0.270, *p* = 0.015; FVC *z*'s: *r* = 0.373, *p* = 0.001; and FEV_1_/FVC ratio *z*'s: *r* = −0.332, *p* = 0.003; Graph 1). FEF_25-75%_*z*'s did not differ between the 2 groups, nor was it correlated to zBMI (*p* = 0.220).

The obese and normal-weight groups differed also in age and height, which led us to compare spirometric values. We did not find any correlation between spirometric *z* parameters and age, but we found a correlation between the FVC *z*'s and FEV_1_/FVC ratio *z*'s versus height (*r* = 0.239, *p* = 0.033; *r* = −0.245, *p* = 0.028, respectively). Therefore, we adjusted our results for those parameters (age and height) and found that both FVC and FEV_1_/FVC ratio *z*'s remained related to zBMI (all *p* < 0.05). This was not the case for FEV_1_*z*'s (*p* > 0.05).

### 3.4. Correlation between HRP and Spirometry Values

All subjects with FEV_1_ (*n* = 8) and/or FVC (*n* = 9) *z*'s below the LLN had AHI ≥ 1 (FEV_1_: *p* = 0.001; FVC: *p* < 0.001). All presented with mild OSAS (1 ≤ AHI < 5) except for one, who had severe OSAS (Graphs 2 and 3). There were more normal-weight subjects than obese subjects with AHI ≥ 1 and FEV_1_*z*'s or FVC *z*'s below the LLN (FEV_1_*z*'s: 6 vs. 2; *p* = 0.003; FVC *z*'s: 6 vs. 3; *p* = 0.010).

Among the three individuals with FEV_1_/FVC ratio *z*′s < LLN, only one had normal AHI indices (*p* = 0.743), and among the five with abnormal FEF_25-75%_*z*'s, two had an AHI ≥ 1 (*p* = 0.881). There was no difference between normal-weight and obese subjects for those parameters.

We did not find any correlation between spirometric data and other polygraphic parameters, such as oAHI, ODI, or mean oxygen saturation, except for FEV_1_*z*'s which were inversely correlated to %time < 90%SpO_2_ (*r* = −0.224, *p* = 0.044). However, %time < 90%SpO_2_ was not significantly different between individuals with normal or abnormal FEV_1_ (FEV_1_*z*'s below the LLN) (0.28 ± 1.1 vs.1.11 ± 2.3; *p* = 0.344).

## 4. Discussion

The first aim of our study was to compare HRP and spirometric values between normal-weight children and adolescents with obesity.

In our study, the proportion of subjects diagnosed with OSAS based on HRP recordings seemed surprisingly low considering that all who were included were suspected of OSAS based on reported snoring, apnea, or diurnal fatigue. On the other hand, the fact that more subjects in the obese group seemed to be affected by OSAS compared with their normal-weight counterparts, despite no difference in HRP recordings between both groups, could be explained by the fact that all subjects were recruited because of suspected OSAS.

The proportion of OSAS in our selected population was also quite low compared to the literature, where it ranges from 46% to 59% [10, 26]. In the general population, obesity has shown to be a significant risk factor for OSAS, associated with a 4.5-fold increased risk [27]. In school-age children with obesity, a prevalence of 44.6% compared with 9.1% in their normal-weight counterparts has been reported, with a relative risk of 4.9 [28].

Regarding our spirometric findings, we found differences between both groups, with higher FVC and lower FEV_1_/FVC ratio *z*'s in children with obesity. FEV_1_*z*'s also seemed higher in the obese group, but the difference disappeared after adjustment for height. Those 3 measurements were strongly correlated to the zBMI (positively for FEV_1_ and FVC and negatively for FEV_1_/FVC ratio *z*'s). It must be highlighted that the spirometric values were within the normal limits in the majority of the children, which was not surprising as we excluded symptomatic asthmatic patients from our study. However, 16% (13/81) of our population had at least one parameter below the LLN, with 11% (9/81) with decreased FVC. Normal-weight children more often had abnormal FEV_1_ and FVC *z*'s, whereas children with obesity seemed to be more affected by low FEV_1_/FVC *z*'s and FEF_25-75%_*z*'s.

In comparison with the literature, we were also confronted with the limitation of the Quanjer GLI 2012 equations that calculate the spirometric *z*'s. Indeed, they are adjusted for age, height, gender, and ethnic group of the subjects, but not for weight or BMI. The strong positive relationship we found between higher FVC and BMI could be due to a difference in puberty onset between the two groups, as in obese children puberty is often more precocious and associated with an earlier growth peak compared with normal-weight children [29, 30]. The difference in puberty development may also explain that at the same age, lung volumes may also be higher in obese subjects. Indeed, whereas child vital capacity increases proportionally faster than FEV_1_, these determinants are temporarily reversed during the adolescent growth spurt. The question therefore arises as to whether the applied correction from Quanjer, which was developed for the general population, is adapted for evaluating children with obesity, as they tend to be taller and physically more mature than their normal-weight counterparts.

Concerning the relationship between spirometric values and weight status, some studies found similar results to ours [5, 7]. Köchli et al. [31] found that in their unselected cohort of 1246 children, FEV_1_ and FVC were significantly higher in obese children, and a decrease in the FEV_1_/FVC ratio was associated with increased BMI, also due to a more important increase in FVC compared to FEV_1_. In their study investigating a population of 327 children and adolescents aged 6-17, Davidson et al. [7] reported a positive linear relationship between FVC (percent predicted) and zBMI and a negative linear relationship between absolute FEV_1_/FVC ratio and zBMI. However, they could not demonstrate a significant relationship between FEV_1_ (percent predicted) nor FEF_25-75_ (percent predicted) and zBMI. Even if our studied population was much smaller, our results compare to others reported and might therefore be considered a representative sample. The fact that known asthmatic patients were excluded in our and Davidson's studies reinforces the importance of our finding a lower FEV_1_/FVC ratio in the obese group, which was mainly due to a higher FVC. Regarding the relationship between asthma and obesity, an official ATS workshop report discussed their relationship extensively [32] and mentioned that airflow obstruction expressed by the FEV_1_/FVC ratio was not usually associated with obesity [33].

Some further studies emphasize that in obese children, FEV_1_/FVC is more reduced than in obese adults [6, 34]. One explanation might be dysanaptic lung growth, e.g., reduced growth of airways relative to lung size, as this seems to be related to, and more common in, obesity. Dysanapsis resulting in expiratory flow limitation could therefore explain the loss of FEV_1_/FVC in children.

In contrast, other and smaller studies [8, 9, 35] did not find any relationship between anthropometric data and spirometric values.

These contrasting results also highlight the inherent difficulty of comparing studies using different spirometric reference values (expressed as absolute, percent predicted, or *z*'s) in different populations (ethnicity, adiposity, age, and with or without respiratory complaints).

The secondary aim of our study was to evaluate the relationship between HRP and spirometric results and weight in this population of children and adolescents with signs and symptoms suspect for OSAS. We did not find any correlation between FEV_1_, FVC, and FEV_1_/FVC ratio *z*'s and AHI, oAHI, or ODI. However, the longer the subjects were below 90% of SpO_2_, the lower their FEV_1_*z*'s, suggesting an obstructive tendency of their airways leading to more pronounced desaturations in case of obstructive respiratory events. Furthermore, subjects with abnormal FEV_1_ and/or FVC *z*'s were all suffering from mild OSAS (AHI > 1) and were mostly of normal weight. The association between abnormal FEV_1_/FVC ratio or FEF_25-75_ and OSAS, or between abnormal FEV_1_*z*'s and %time < 90%, was not so clear, probably due to the small number of subjects concerned. This limitation probably also prevented us from detecting a difference between the groups, although obese subjects did seem more represented.

When reviewing the literature, a recent pediatric study by Van Eyck et al. [11], performed in a much larger population of 185 children who were either overweight or obese (mean *z*'s 2.4 in the range of 1.5-3.6), showed a significant decrease of all measured lung function parameters in subjects with moderate-to-severe OSAS compared to children without OSAS. FEV_1_ and FVC were inversely correlated with the severity of OSAS, independent of the degree of adiposity for FEV_1_. However, no correlations were found for the FEV_1_/FVC ratio after correction for zBMI. The authors suggested that pulmonary function abnormalities could participate in the pathology of OSAS and that improving lung function might diminish the severity of OSAS. Although it is difficult to compare studies because of differences in OSAS definitions and type of pulmonary function tests realized (body plethysmography or spirometry only), we could explain the lack of relationship between spirometric and polygraph values and weight in our study by the fact that we had a much smaller study population compared to the Van Eyck study and that the majority of our subjects suffered from only mild OSAS. Indeed, in subjects with more severe OSAS, such as in adult studies, the results are more consistent, with a clear link between lung function and OSAS severity [36–38]. The fact that our subjects with abnormal spirometric results were all suffering from mild OSAS may suggest that with a larger sample, we would also have found a correlation between those 2 entities. To our knowledge, no studies have explored this relationship in children with normal weight before us. In subjects who suffered from OSAS, anomalies in pulmonary function were more frequent, which leads us to suggest that this group is at greater risk of concomitant pulmonary involvement and therefore should benefit from screening.

### 4.1. Limitations

We studied only spirometry but measured no further lung volumes, such as total lung capacity (TLC) and functional residual capacity (FRC). Correlations between these volumes have often been associated with obesity in the literature. Furthermore, as already stated, ours was a small study population where few subjects suffered from OSAS and mostly in a mild form, thereby limiting our capacity to draw stronger conclusions. Finally, the cross-sectional nature of our analysis limits our interpretations, and further observational or interventional studies are needed to clarify the relationships.

## 5. Conclusion

When comparing children and adolescents with normal weight to subjects with obesity, the prevalence of OSAS was similar, whereas spirometric values were strongly related to zBMI, probably due to advanced puberty coupled with an accelerated growth spurt in obese children. FEV_1_ was more frequently abnormal in normal-weight subjects, while obese subjects presented low FEV_1_/FVC and FEF_25-75%_*z*'s. Our result might also suggest that children presenting an obstructive lung function may also suffer from more pronounced desaturation during sleep. Moreover, all subjects with abnormal spirometric values were suffering from at least mild OSAS, which was again more frequent in normal-weight subjects. Obesity and OSAS may share the same pathophysiology, which could be a manifestation of the general inflammation commonly seen in obesity. However, contrary to what we expected, normal-weight children seemed more at risk of presenting pulmonary limitations when suffering from OSAS than subjects with obesity. Much larger studies are needed to confirm our results.

## Figures and Tables

**Table 1:** Characteristics of subjects.

	Normal weight *N* = 30	Obese *N* = 51	*p*
Gender: female: *n* (%)	15 (50.0)	20 (39.2)	0.344
Age (years)	7.4 ± 2.4	10.7 ± 3.5	<0.001^∗^
*Anthropometrics*			
Weight (kg)	23.9 ± 8.2	61.0 ± 27.8	<0.001^∗^
Height (cm)	123.4 ± 16.0	146.4 ± 19.8	<0.001^∗^
BMI *z*-score	−0.4 ± 0.9	2.8 ± 1.1	<0.001^∗^
*Spirometric measurements*			
FEV_1_ (*z*-score)	−0.55 ± 0.93	0.10 ± 1.14	0.010^∗^
FVC (*z*-score)	−0.80 ± 0.99	0.06 ± 1.06	0.001^∗^
FEV_1_/FVC (*z*-score)	0.62 ± 0.93	−0.04 ± 0.96	0.003^∗^
FEF_25-75%_ (*z*-score)	0.18 ± 1.00	−0.15 ± 0.99	0.153
FEV_1_ (% of predicted)	93.2 ± 11.8	98.7 ± 17.1	0.120
FVC (% of predicted)	90.0 ± 12.6	99.0 ± 17.2	0.015^∗^
FEF_25-75%_ (% of predicted)	74.7 ± 47.0	66.1 ± 42.5	0.401
Below the LLN values			
FEV_1_*z*'s *n* (%)	6 (20%)	2 (4%)	0.019^∗^
FVC *z*'s *n* (%)	6 (20%)	3 (6%)	0.055
FEV_1_/FVC *z*'s *n* (%)	0	3 (6%)	0.171
FEF_25-75%_*z*'s *n* (%)	1 (3%)	4 (8%)	0.415
*Home respiratory polygraphy parameters*			
Mean oxygen saturation (%)	96.9 ± 0.8	96.7 ± 1.2	0.233
%time < 90%SpO_2_	0.26 ± 0.8	0.43 ± 1.5	0.576
ODI (events/h)	2.9 ± 6.2	4.1 ± 8.2	0.499
oAHI (events/h)	0.5 ± 1.5	0.2 ± 0.6	0.373
AHI (events/h)	2.4 ± 5.6	2.1 ± 4.5	0.766
AHI ≥ 1: *n* (%)	11 (36.7)	24 (47.1)	0.362
AHI ≥ 5 : *n* (%)	4 (13.3)	5 (9.8)	0.625
OSAS severity			0.613
No OSAS (AHI < 1): *n* (%)	19 (63.3)	27 (52.9)	
Mild OSAS (AHI: 1-4): *n* (%)	7 (23.3)	19 (37.3)	
Moderate OSAS (AHI: 5-9): *n* (%)	2 (6.7)	2 (3.9)	
Severe OSAS (AHI: ≥10): *n* (%)	2 (6.7)	3 (5.9)	

Abbreviations: BMI: body mass index; FEV_1_: forced expiratory volume in 1 second; FVC: forced vital capacity; FEF_25-75%_: forced expiratory flow at 25-75% of forced vital capacity; LLN: lower limit of normal; ODI: oxygen desaturation index; oAHI: obstructive index; %time < 90%SpO_2_: percentage of analyzed time under 90% of partial oxygen saturation (%); AHI: apnea-hypopnea index. Spirometric measurement *z*-score and % predicted values calculated using GLI 2012 equations. Data are presented as the mean ± SD. ^∗^*p* < 0.05.

## Data Availability

The data used to support the findings of this study are available from the corresponding author upon request.

## Supplementary Materials

Supplementary 1 Graph 1: linear regression between FEV_1_, FVC, FEV_1_/FVC ratio, and FEF_25-75%_*z*-scores versus BMI *z*-score.

Supplementary 2 Graph 2: FEV_1_*z*-score values and their relationship to OSAS severity. Dotted line: LLN (FVC *z*-score < −1.64).

Supplementary 3 Graph 3: FVC *z*-score values and their relationship to OSAS severity. Dotted line: LLN (FVC *z*-score < −1.64).
