# Supplementary figures and images for: Home Respiratory Polygraphy and Spirometry in Normal Weight and Children with Obesity Suspected for Obstructive Sleep Apnea Syndrome: Are There Any Associations?
Source: Pulm Med. 2023 Jan 31;2023:1532443. doi: 10.1155/2023/1532443 (PMC9906030; doi:10.1155/2023/1532443)

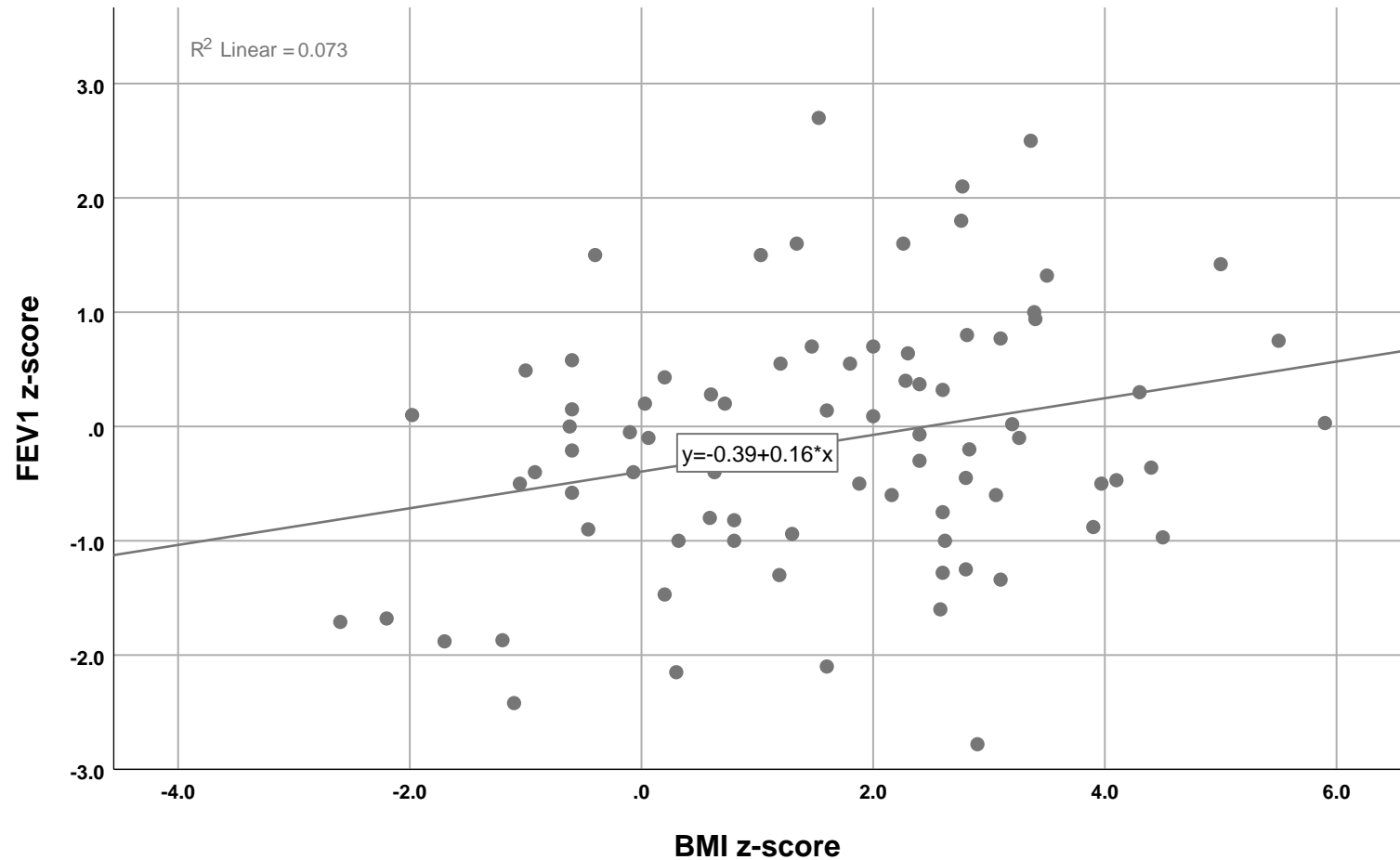

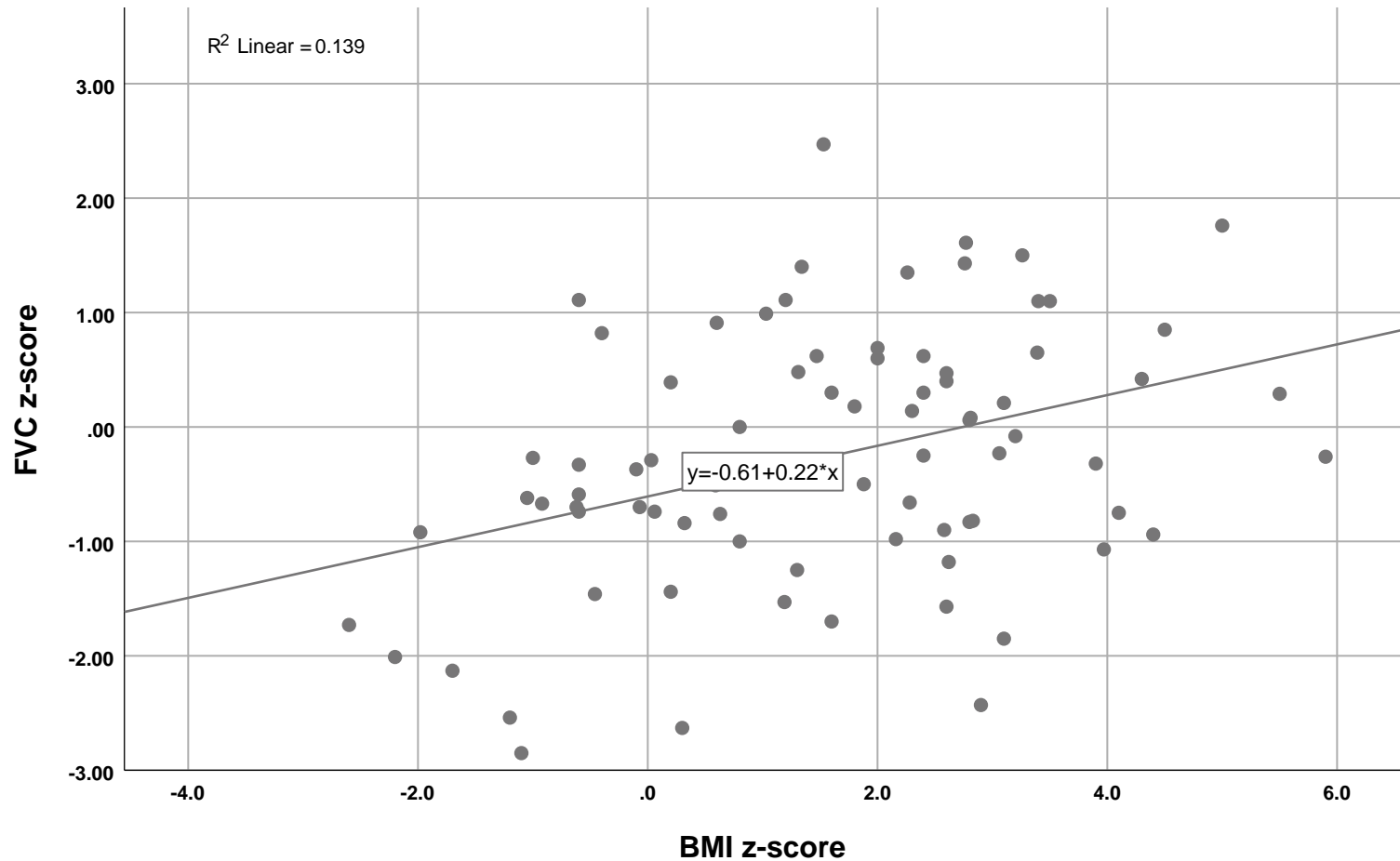

FEV1/FVC z-score

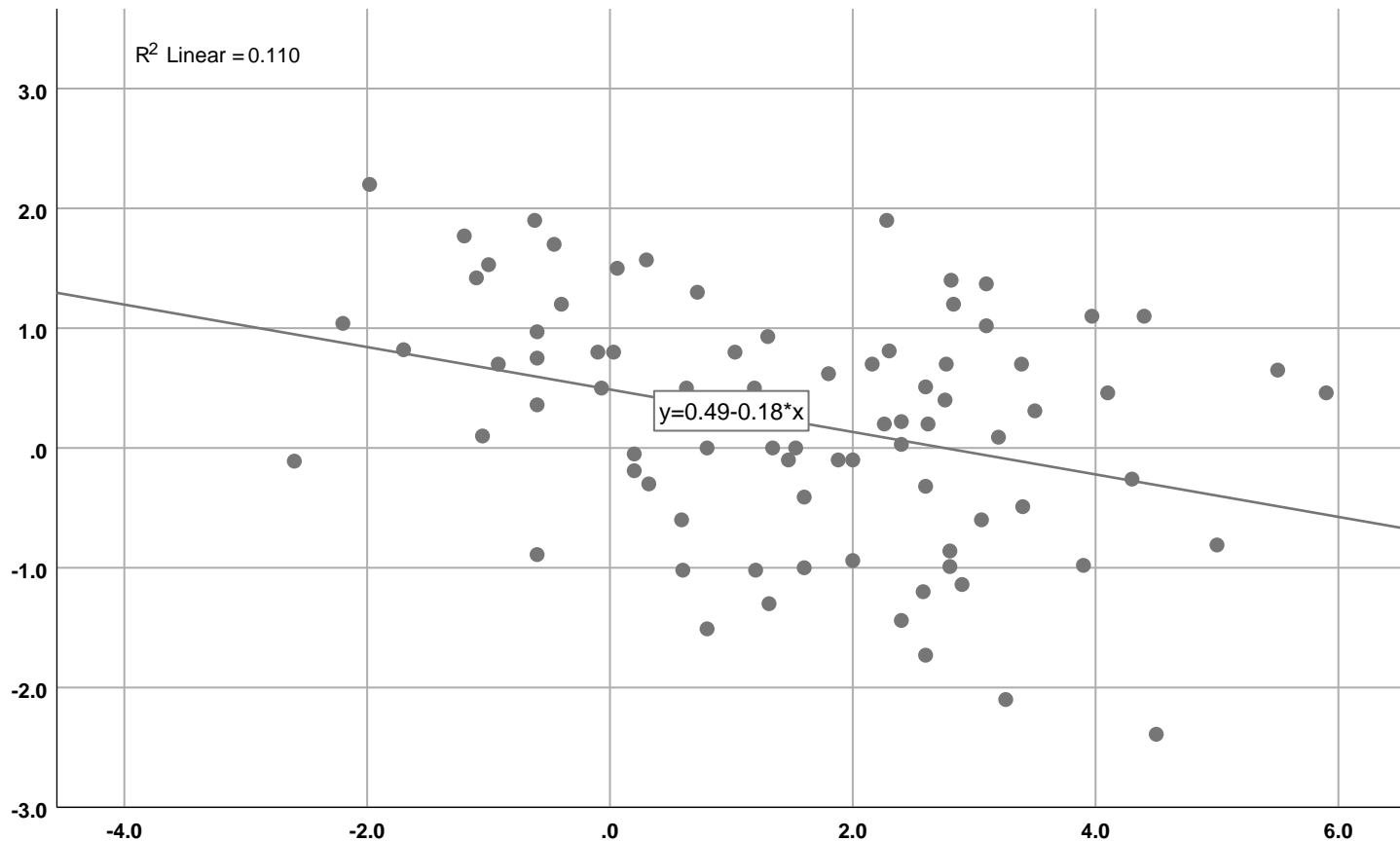

BMI z-score

FEF 25-75 z-score

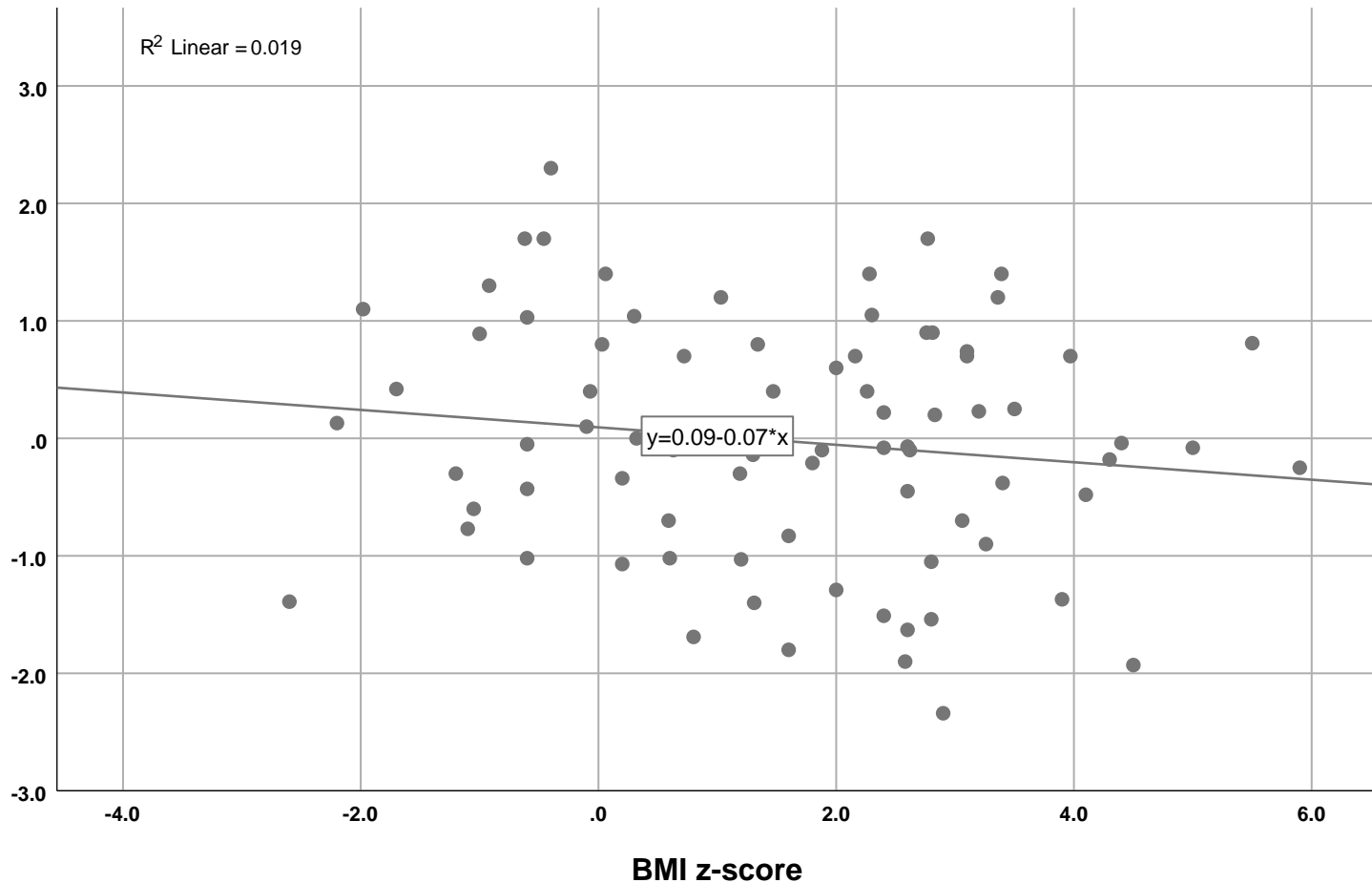

Supplement: Supplementary 1 — Graph 1: linear regression between FEV1, FVC, FEV1/FVC ratio, and FEF25-75%z-scores versus BMI z-score. [file 1532443.f1.pdf]

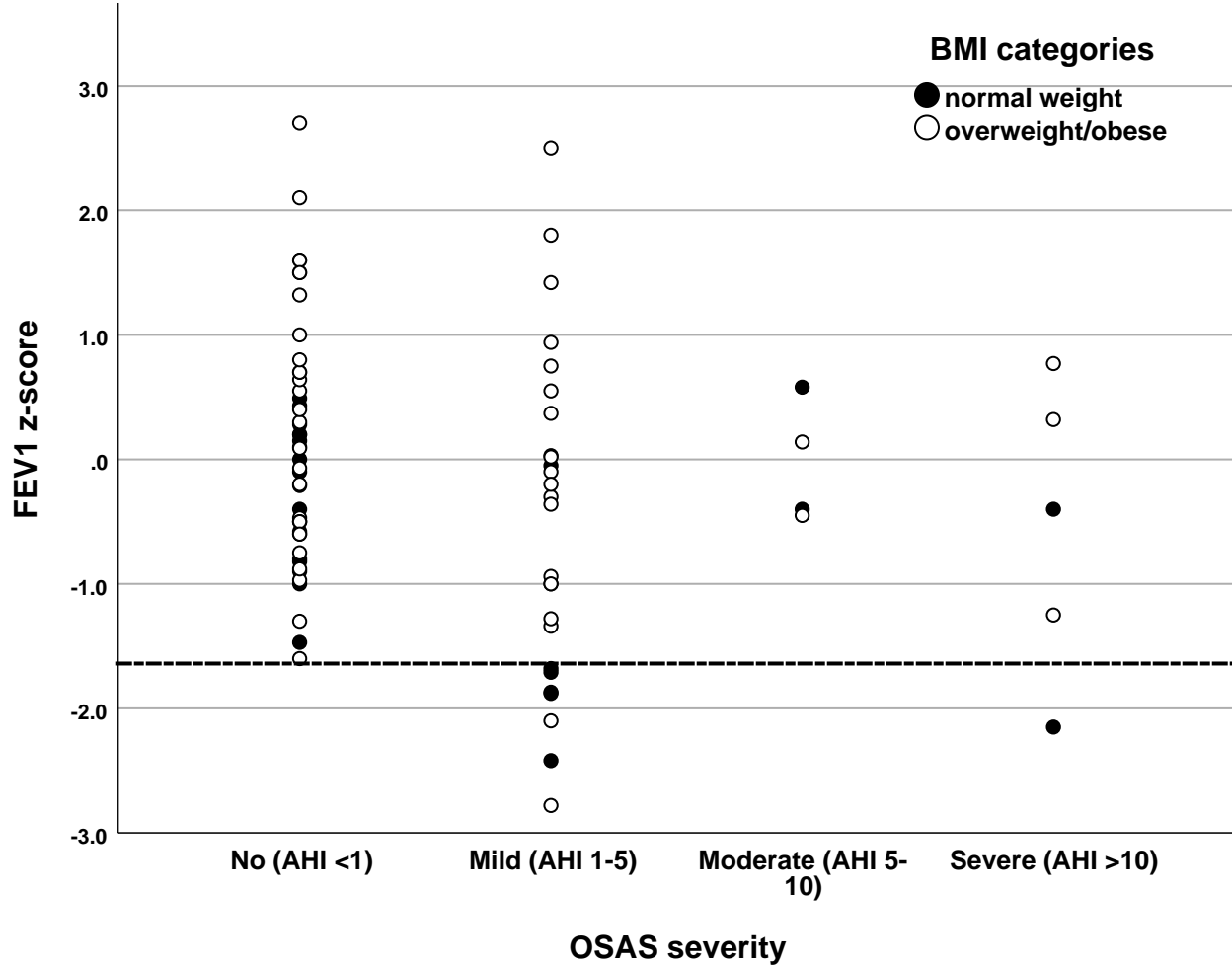

Supplement: Supplementary 2 — Graph 2: FEV1z-score values and their relationship to OSAS severity. Dotted line: LLN (FVC z-score < −1.64). [file 1532443.f2.pdf]

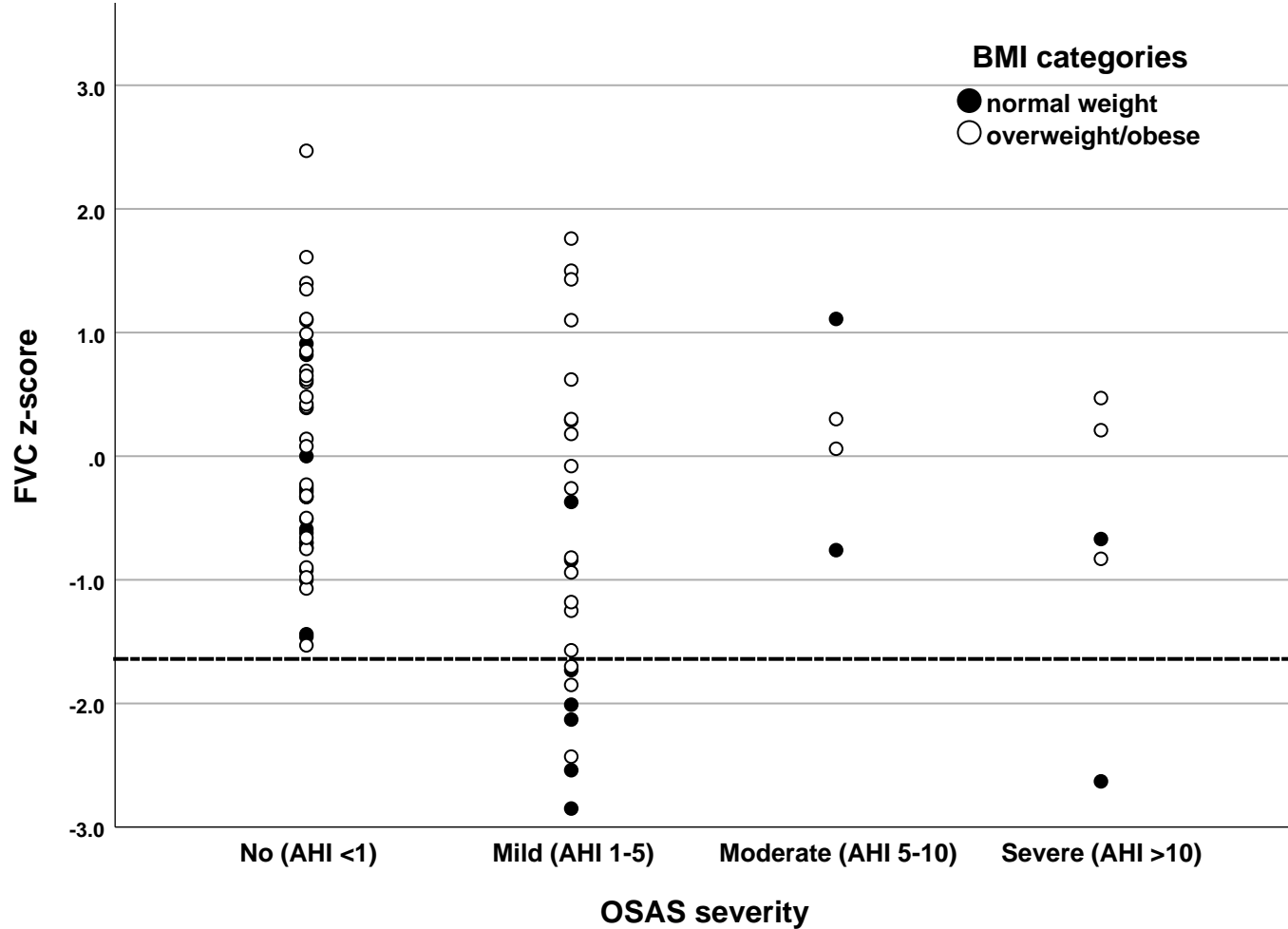

Supplement: Supplementary 3 — Graph 3: FVC z-score values and their relationship to OSAS severity. Dotted line: LLN (FVC z-score < −1.64). [file 1532443.f3.pdf]
